# Overview and experience of the YODA Project with clinical trial data sharing after 5 years

**DOI:** 10.1038/sdata.2018.268

**Published:** 2018-11-27

**Authors:** Joseph S. Ross, Joanne Waldstreicher, Stephen Bamford, Jesse A. Berlin, Karla Childers, Nihar R. Desai, Ginger Gamble, Cary P. Gross, Richard Kuntz, Richard Lehman, Peter Lins, Sandra A. Morris, Jessica D. Ritchie, Harlan M. Krumholz

**Affiliations:** 1 Section of General Medicine, Department of Medicine, Yale School of Medicine, New Haven, CT, USA; 2 National Clinician Scholars Program, Yale School of Medicine, New Haven, CT, USA; 3 Department of Health Policy and Management, Yale School of Public Health, New Haven, CT, USA; 4 Center for Outcomes Research and Evaluation, Yale–New Haven Hospital, New Haven, CT, USA; 5 Johnson & Johnson, New Brunswick, NJ, USA; 6 Janssen Pharmaceutical Companies of Johnson & Johnson, High Wycombe, UK; 7 Section of Cardiovascular Medicine, Department of Medicine, Yale School of Medicine, New Haven, CT, USA; 8 Cancer Outcomes, Public Policy, and Effectiveness Research (COPPER) Center and Yale Cancer Center, Yale School of Medicine, New Haven, CT, USA; 9 Medtronic, Inc., Minneapolis, MN, USA; 10 Stonebank Medical Ltd., Banbury, UK

**Keywords:** Medical research, Research data

## Abstract

The Yale University Open Data Access (YODA) Project has facilitated access to clinical trial data since 2013. The purpose of this article is to provide an overview of the Project, describe key decisions that were made when establishing data sharing policies, and suggest how our experience and the experiences of our first two data generator partners, Medtronic, Inc. and Johnson & Johnson, can be used to enhance other ongoing or future initiatives.

## Introduction

Remarkable quantities of participant-level data and aggregate-level results are generated through clinical research, much of which is never published or disseminated, limiting its contribution to current knowledge and practice. Over the past five years, many leaders within the clinical research enterprise have made bold statements and adopted policies that promote clinical research data sharing, defined by the National Academy of Medicine (formerly known as the Institute of Medicine of the U.S. National Academy of Sciences) as the distribution of individual participant-level clinical trial data to researchers based outside the original study investigator team to enable independent use for scientific purposes^1^. Examples of organizations making recommendations to facilitate data sharing include the National Academy of Medicine^1^, the World Health Organization (http://www.who.int/medicines/ebola-treatment/data-sharing_phe/en/), the U.S. National Institutes of Health (NIH)^2^, the European Clinical Research Infrastructure Network^3^, and the International Committee for Medical Journal Editors^4^, as well as both PhRMA and EFPIA (https://www.phrma.org/press-release/joint-efpia-phrma-principles-for-responsible-clinical-trial-data-sharing-become-effective-today), the pharmaceutical trade organizations representing manufacturers in the United States and European Union, respectively.

Given already widespread, and still increasing, support for data sharing^5–7^, the challenge facing the field now is to develop fair and sustainable approaches for investigators to access these data and utilize them to advance science. The Yale University Open Data Access (YODA) Project, an initiative housed within Yale University, has been actively working to facilitate access to clinical trial data since 2013. The purpose of this article is to provide an overview of the Project, describe key decisions that were made when establishing data sharing policies, and suggest how our experience and the experiences of our first two data generator partners, Medtronic, Inc. and Johnson & Johnson, can be used to inform and thereby enhance other ongoing or future initiatives.

## Results

### Overview

In 2011, the YODA Project was founded to promote data sharing among the scientific community and develop a platform that could be used as a means of responsible data sharing^8^. At its inception, the Project established an organizing mission to guide its decision-making: 1) promote the sharing of clinical research data to advance science and improve public health and healthcare; 2) protect the rights of research participants; 3) promote the responsible conduct of research; and 4) ensure good stewardship of clinical research data by external investigators.

The YODA Project began as part of a partnership with Medtronic, Inc. in 2011 with two purposes^9,10^. First, the Project was tasked with soliciting two independent analyses of individual participant-level data (IPD) from all published and unpublished trials relating to its marketed product recombinant human bone morphogenetic protein-2 (rhBMP-2). Second, following completion of these analyses in 2013, the data were to be made available for sharing with the broader scientific community. This partnership (and the data sharing) concluded in 2015.

In 2014, the YODA Project began a partnership with Johnson & Johnson to develop and implement a broadly-encompassing policy to share clinical trial data for all non-Phase I interventional trials of the company’s pharmaceutical products; data were first made available to external investigators through this initiative in October 2014^11^. The scope of this agreement was expanded in 2015 to include trials of medical device products and again in 2017 to include trials of consumer products used by health authorities for approval from 2014 onwards; the detailed policy scope is publicly available (http://yoda.yale.edu/policies-procedures-guide-external-investigator-access-clinical-trial-data).

In 2016, the YODA Project began a partnership with SI-BONE, Inc., a smaller medical device company, to share clinical trial data relating to its marketed product, the iFuse sacroiliac joint fusion implant system.

### Policy Development Process

**Figure 1:**
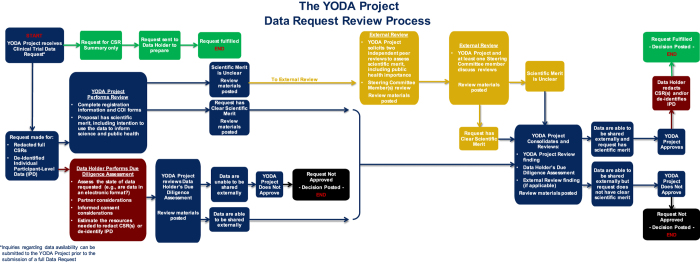
The YODA Project data request review process.

The YODA Project data sharing policy was initially established for access to Medtronic’s rhBMP-2 clinical trial data, and later for access to Johnson & Johnson’s broad portfolio of clinical trial data. The policy established the procedures external investigators were required to follow to gain access to data for independent scientific examination (see Fig. 1 for illustration). Development was iterative and informed by the following:

- Input from partnering companies that have generated the clinical trial data;
- Input from the YODA Project’s Steering Committee, an independent group of leaders in the fields of clinical research and biomedical ethics assembled by the YODA Project to provide guidance;
- Input from other experts in the field, industry, regulators, and the general public through a public comment period and personal communication;
- Review of the literature and policies from other organizations engaged in clinical trial data sharing, such as the NIH’s Biologic Specimen and Data Repository Information Coordinating Center (BioLINCC) within the U.S. National Heart, Lung, and Blood Institute^12,13^; and
- The experience gained by the YODA Project sharing IPD with external investigators.

### Key Aspects of the YODA Project Data Sharing Policy

There are several key aspects that were considered as decisions were made when establishing the Project’s data sharing policy. These decisions, and the lessons we learned from their implementation, can be used to inform and enhance other ongoing or future data sharing initiatives.

#### Commitment to Transparency

Transparency enhances trust in the integrity of the data sharing process and the resulting research, as well as clarity of parties involved. To ensure transparency of the overall effort, the YODA Project makes as much information publicly accessible as possible, including Project personnel; requirements for data access; information on trial data available, such as Clinical Study Report summaries and hyperlinks to registration records on ClinicalTrials.gov and publications on PubMed.gov; information on all submitted proposals for data, such as the number approved and rejected; and approved research proposals in their entirety, including the resulting research upon completion. In addition, all major decisions made by the YODA Project have included an opportunity for public comment, including the finalized policies and procedures for making clinical trial data available.

#### Full Authority and Independence

Data sharing requires mutual trust and collaboration with each partnering company, while at the same time upholding the independence and impartiality of the data-sharing organization. The YODA Project requires that it has full decision-making authority over the release of the data and serves as an independent intermediary to manage requests and promote data use. The final decisions regarding the design of the data request process, the criteria for access, and approval or rejection of requests all reside with the YODA Project. Maintaining this final authority is intended to build trust in the process and reduces opportunities for real or perceived influence.

#### Independent Steering Committee

The YODA Project assembled a Steering Committee of external experts, an independent group of leaders in the fields of clinical research and biomedical ethics, to provide guidance as it developed standards, policies, and procedures. The YODA Project was able to call on the Committee’s expertise to inform the data release process, including how best to make trial information available, what data request requirements should be established, and what the nature of the data request review process should be. The Committee also provided valuable feedback on other issues, such as the importance of making meta-data available (i.e., information about the trials being shared), including statistical analysis plans, blank case report forms, and study protocols, along with more difficult issues such as what to do when meta-data materials had not been prepared in English. In addition, the Committee assisted in making sure that the procedures were consistent with the standards of ethical research, including avoidance of conflict of interest and protection of patient privacy.

#### List of Available Trials

Publicly listing trials that are available to external investigators is crucial both to promoting use of the shared data and establishing the transparency of the initiative. However, the proactive preparation of a catalogue of available trials for a large company with a multitude of marketed products is time and resource intensive. Therefore, to ensure the efficient use of resources, at the initiation of our data sharing partnerships, the criteria for defining trials in-scope for sharing were established. Pertinent to this decision is the protection of patient privacy, as data for which the privacy and confidentiality of research participants cannot be protected should not be routinely shared, an important consideration for studies of rare diseases or those that have few participants. Furthermore, for any product, determinations need to be made as to whether the data are owned solely by the company, as medical products are frequently jointly owned or marketed, requiring consent from both manufacturers before the data could be shared.

For the partnership with Johnson & Johnson, the number of trials in-scope was large, particularly because of the company’s commitment to make older trials available. Thus, at the outset, a subset of contemporary trials for commonly used products likely to be of interest to medical researchers were identified and publicly listed. Because this list was not exhaustive of all trials that could be made available, the Project also established a method for external investigators to inquire about the potential availability of other trials that were not listed. As additional trials were requested and made available, they were added to the public listing. Further, to facilitate identification of trials by investigators, the listing was organized into multiple views, including by product, therapeutic area, and condition studied, and also made searchable on key data elements, such as enrollment and trial demographic characteristics.

#### Supporting Documentation

In order to increase the likelihood that the data can be used for research, interested parties need full understanding of the data resources being made available. To this end, supporting documentation and materials, or meta-data, are described for each available trial, including hyperlinks to the ClinicalTrials.gov registration (or a number from another international registry) and known trial publications through PubMed.gov. Similarly, documentation, such as blank case report forms, clinical study reports, data definition specifications, protocols with amendments, and/or statistical analysis plans, enables investigators to more efficiently and accurately determine for what purpose the data can be best used if access is obtained. Supporting documentation materials made publicly available are listed in Box 1.

#### Research Proposal Submission and Public Posting

It is essential to demonstrate that research by external investigators making use of data made available by industry is responsibly conducted, since concerns continue to be voiced about the potential for its misuse and misinterpretation^6^. To promote the responsible conduct of research, the YODA Project adopted a controlled access model^14^, requiring investigator registration and submission of a proposal to be reviewed prior to approval, which can then be subsequently publicly posted once data access is granted. Specific information must be submitted, including the principal investigator, key project personnel, and the research proposal; requirements are listed in Box 2. Notably, a statistician is not required to be included among key project personnel. Requiring registration and public posting of proposals potentially fosters collaboration and open science, while also making it easier for interested independent scientists to evaluate research using shared data.

#### Blinded Request Review by the YODA Project

All data requests undergo review by multiple clinical investigator members of the YODA Project, blinded to all identifying details about the investigator, including funding source. Review helps to ensure that the proposal has scientific merit, in that 1) the scientific purpose is clearly described; 2) the data requested will be used to create or materially enhance generalizable scientific and/or medical knowledge to inform science and public health; and 3) the proposed research can be reasonably addressed using the requested data. The assessment of whether the proposed research can be reasonably addressed using the requested data includes evaluating whether the variables needed for the proposed analysis are included in the requested data and whether the question could best be addressed with either individual participant-level or summary-level data. While the YODA Project review is not a detailed technical evaluation of the proposed research *per se*, a high-level review evaluates whether the proposed statistical methods can answer the scientific question. The review process permits peer-review feedback and/or requests for clarification prior to approval determination. All YODA Project reviews are publicly posted.

There have been instances where the YODA Project has also provided feasibility feedback to facilitate the use of data. For instance, one request was received for a methodological study characterizing trial populations’ representativeness that would use all trials listed on the YODA Project website (there were 123 at the time); it was determined that preparation of these data would take upwards of 6 months. The YODA Project communicated this to the investigator, who decided to narrow their proposal to use only those trials that were already de-identified and prepared for sharing and planned to subsequently amend their request as more data became available.

#### Blinded Request Review by Partnering Company

All data requests undergo a due diligence assessment by the partnering company, a blinded assessment of whether the data are already prepared or need to be prepared in a format that is de-identified and can be made available to external researchers, as well as whether the variables of interest are available and to check whether a similar analysis is underway or has already been completed by the company; if so, this information is shared with the investigator. For example, the YODA Project received a data request that proposed characterizing reasons for trial eligibility screening failures in late phase trials in advanced genitourinary cancers. After examining the data, the partnering company advised the YODA Project that reasons for screening failure were not documented for all occurrences. After relaying this information to the investigator, they still chose to proceed with their proposal. All company due diligence assessments are publicly posted.

#### Opportunity for Collaboration with Partnering Company

Data sharing initiatives create a means for investigators to conduct independent analyses, but they also provide opportunities for new collaborations.^4,15^ To foster these collaborations, the YODA Project established a process that allows for and coordinates communication between investigators and the partnering company, when desired by the investigator. This process allows external investigators to be made aware of any similar ongoing research efforts and potentially foster collaboration when there is mutual interest.

#### Data Use Agreement

All approved data requests require a signed Data Use Agreement (DUA) between Yale University, representing the YODA Project, and the affiliated institution representing the external investigator. The DUA is intended to ensure that the investigator will protect the confidentiality of the data, will not attempt to re-identify trial participants, and will not copy, retransmit, or use the data in any manner other than for the purpose described in the data request. A template is publicly available for investigators to review prior to requesting data (http://yoda.yale.edu/data-use-agreement). The DUA limits data access to one year, ensuring frequent updates and contact between the investigators and the YODA Project, as the agreement can be re-approved for additional time if the research is ongoing. The DUA also specifies that data cannot be used for non-scientific purposes, such as in pursuit of litigation or for commercial interests. While there remains ambiguity in what constitutes non-scientific use, and commercial use in particular, the agreement is intended to promote a good faith approach among researchers. Moreover, the DUA requires investigators to report any notable safety results to the partnering company, as it is the legal responsibility of any manufacturer of all U.S. Food and Drug Administration regulated medical products to report these findings to appropriate regulatory officials. Finally, to ensure that investigators understand the DUA, the YODA Project developed a training module that must be completed prior to submission of an investigator’s first data request, emphasizing important points and policies governing access.

#### Secure Data Access or Transfer

The YODA Project has employed two different models of controlled data access for approved investigators, adopted to ensure the security and prevent wider distribution of shared clinical trial data. In our partnership with Medtronic, password-protected, de-identified data were distributed using secure file transferring services to approved investigators. Certificates of data destruction were required at DUA expiration. This method is not costly and offers greater flexibility to investigators and enables use of additional software types (so long as the investigator has his/her own license) and linkage to other data sources, but increases the risk of unapproved distribution.

In the YODA Project partnership with Johnson & Johnson, the company entered into licensing agreements with a secure data sharing platform allowing virtual data access to credentialed and approved investigators while precluding data download or distribution. While secure, these platforms are expensive and limited to the licensed analytic tools and software. Moreover, there have been challenges to uploading complementary or other trial data onto the licensed platform, preventing the combination of data from different sources, such as for meta-analyses, so that investigators had to rely on the aggregate-level data. Lastly, users of the platform have also reported it to be difficult to navigate, time intensive to learn, and noted that the programs occasionally closed, causing loss of work and the user to be logged out. Nevertheless, on the whole, these platforms have offered a reasonable technical means for data security and protection against redistribution.

There are certain circumstances that may require access to clinical trial data outside of the secure platform, such as the need to use proprietary software that cannot be placed in the platform or to pool data from other sources that cannot access the platform. However, given the sensitivity of patients’ trial data, including the importance of protecting privacy and risk of re-identification, as well as the greater risk for unauthorized data dissemination and analysis, access outside of the secure platform requires clear justification. In collaboration with Johnson & Johnson, the YODA Project developed an exception request process, in which the investigator is required to provide additional information to support his/her request for direct access to the data. This includes 1) strong rationale for why the requested data can uniquely be used to address the proposed project aims, and reasons why other clinical research data are not available or cannot be used; 2) reasons why the platform may not permit the proposed analyses; and 3) a description of the protections in place to ensure data security outside of the platform, including technological and related procedural safeguards. Based on this information, the YODA Project then assesses the need for data access outside of the secure platform. Not all exception requests are approved.

#### Results Dissemination

Upon project completion, the YODA Project requires public dissemination of research findings, preferentially through the peer-reviewed biomedical literature or at scientific meetings. This explicitly promotes the scientific process and peer review, ensuring the methods used meet the minimal scientific standards prior to dissemination. Once the proposed research has been publicly disseminated through peer-reviewed publication, these findings can be further discussed via non-peer-reviewed forums, including internet posts, newspaper articles, or other means. Regardless of whether the work is published, all results from analyses are required to be reported back to the YODA Project in summary form to be publicly posted at the expiration of the DUA, including whether or not the project was ultimately completed, ensuring public transparency and accountability.

Prior to publication or presentation at a scientific meeting, copies of any abstract or manuscript generated from the data request are required to be shared with the YODA Project. This helps to track whether projects are progressing, clarifies what new scientific information has been generated, and updates our records of the completion and publication of analyses, informing future data sharing efforts.

#### Data Access Fee

Thus far, the YODA Project and its data partners have not imposed a fee for investigators to access data through the YODA Project. The entire cost has been covered by the industry partners. However, the sustainability of data sharing initiatives, and their current reliance on industry funding, will likely require reconsideration of funding models, especially given the hope that data sharing efforts will broaden to include smaller pharmaceutical, medical device, and biotech companies, as well as academic institutions. A solution may be for investigators to apply for research grants from government agencies or non-profit organizations to support the use of shared data. Similarly, funding to prepare data for external sharing should be built into grants awarded to academic groups when they conduct their own clinical trials. In the current environment, in which funding is already very competitive, whether such funding models would work is uncertain.

### Medtronic Experience

As noted above, Medtronic’s rhBMP-2 clinical trial data were shared once the two independent systematic reviews and meta-analyses of the data had been published, which occurred in June 2013^16–18^. Concurrently, an online application process was established and a total of 4 requests for the rhBMP-2 trial data were received. All 4 were approved (additional details, including the protocols, can be found at http://yoda.yale.edu/medtronic-past-data-recipients), 2 of which were completed and resulted in peer-reviewed publications^19,20^. While the rhBMP-2 trial data were generated by a single company, individual trials did not adhere to the same data formatting and standards, requiring Medtronic to first invest resources into data preparation for sharing, as well as for investigators to review data files and recode data as needed to allow aggregation and meta-analysis. The remaining 2 were not completed, both due to investigator commitments to other projects and lack of time. No instances of data redistribution were reported and certificates of data destruction were received for the 2 uncompleted projects; the 2 completed projects retain access to the data for 5 years in the event that questions are raised about their published analyses, after which time the DUA expires. Because no requests for the data were received after January 2014, Medtronic discontinued making the data available in summer 2015.

### Johnson & Johnson Experience

Johnson & Johnson began sharing clinical trial data for all trials of the company’s pharmaceutical products in October 2014 and later expanded its scope to include trials of medical device and consumer products. While the company is willing to make all non-Phase I interventional pharmaceutical trials available, at the initiation of the partnership, we proactively identified contemporaneous trials likely to be of greatest interest to the scientific community and listed them on the YODA Project website. At the same time, the inquiry process was critical during the early days of the partnership; as of August 2018, 161 inquiries for more than 200 unique trials have been submitted, identifying 124 that could be made available for sharing (Table 1). Most common reasons for unavailability include that the trial was out-of-scope (i.e., phase 1 healthy volunteer studies), ongoing or completed less than 18 months ago, or that regulatory approval had not yet been received (details are available at: http://yoda.yale.edu/request/summary-data-inquiries-and-requests/details-inquiries-submitted-data-not-yet-available). However, it should be noted that when investigators inquire about the availability of an out-of-scope trial, they are invited to submit an abstract that conveys the scientific importance of the planned research, which is then evaluated by the YODA Project on a case by case basis; thus far, only 1 investigator has followed up with an abstract. As of August 2018, 270 clinical trials are listed on the YODA Project website, along with supporting documentation (Table 2), and additional details, including specific therapeutic areas (Table 3), specific conditions studied (Table 4 (available online only)), specific products (Table 5), and specific product classes (Table 6). Johnson & Johnson started to adopt Clinical Data Interchange Standards Consortium (CDISC, https://www.cdisc.org/) in 2001 and by 2003 study data sets were being routinely reported in CDISC format. By using CDISC standards, it brings benefits in enabling the high reuse of software for data analysis. It also minimizes the time and effort of data preparation before data sharing can commence. For the researcher, it increases familiarity of data structures and helps to ensure consistency between trials (and even sponsors).

As of August 2018, 100 data requests have been received from 89 unique principal investigators for a median of 3 trials per request (Interquartile Range, 1-9), 90 (90.0%) of which have been approved with a DUA signed or in progress, 2 (2.0%) remain under review pending revision, and 8 (8.0%) were withdrawn or closed (Table 7). Withdrawals generally occurred because the due diligence assessment determined that the data needed to address the proposed question were not available (such as requests for pharmacokinetic data or endoscopic video data) or because special statistical software was needed that could not be imported into the secure data sharing platform. Notably, two withdrawn requests resulted in direct collaboration with Johnson & Johnson to pursue the research. No request has been rejected, although 36 (36.0%) of submitted research proposals required revision after YODA Project review for clarification or elaboration, 2 of which were never resubmitted and are now considered withdrawn.

Among the 270 clinical trials currently listed on the YODA Project website, 183(67.8%) have thus far been requested, although 46 have only been available since January 1, 2018. The most common purposes of the proposed projects include (not mutually exclusive) addressing secondary research questions (n = 57; 57.0%), combining data as part of larger meta-analysis (n = 45; 45.0%), and validating previously published studies (n = 24; 24.0%).

Of 82 approved requests provided data access, most remain in progress; 11(13.4%) have at least one publication^21–31^ (12 publications in total, see Table 8 for publications list) and 7 (8.5%) have at least one article under peer-review, whereas 19 (23.2%) have been discontinued, either because of investigator commitments to other projects and lack of time or because the investigator did not have sufficient statistical expertise to conduct analyses within the secure data sharing platform. All of the projects that have been submitted for publication described analyses representing those specified in the original research proposal. Finally, no instances of data redistribution have been reported.

## Discussion

Data sharing and data transparency are quickly becoming the new standard in pharmaceutical and medical device science and in clinical research more broadly. Many national and international organizations are adopting policies to advance scientific and medical knowledge through data availability and transparency that will ultimately improve public health and healthcare delivery, advancing scientific understanding of disease diagnosis and prognosis through the development of novel tools and approaches, while also improving existing knowledge of treatment safety and efficacy.

The early experiences of the YODA Project can be used to inform the field and other data sharing initiatives. Certain decisions contrast with other existing clinical trial data sharing initiatives (including those found at https://biolincc.nhlbi.nih.gov/studies/, https://ClinicalStudyDataRequest.com/, and https://dcri.org, as examples). For instance, the decision to *reactively* de-identify data for sharing in response to requests has meant that 68% of trials listed on the YODA Project website have been used, as opposed to platforms that have *proactively* de-identified data for sharing reporting that approximately 15% of listed trials having been used^32,33^. Similarly, while the direct, secure data transfer used for Medtronic rhBMP-2 trials was simple, the secure platform used for Johnson & Johnson’s trials is more challenging for investigators, particularly those without advanced statistical expertise. Nevertheless, investigator experience with this platform mirrors the experience of those using data shared through the U.S. National Heart, Lung, and Blood Institute BioLINCC repository^34^. Lastly, Johnson & Johnson makes all non-Phase I interventional pharmaceutical trials available, including older trials, whereas many current initiatives and company policies are focused on sharing clinical trial data as of a specific date going forward, limiting the availability of trials that examined medical products commonly being used by patients today.

Outstanding issues remain for the field to address, including how to make older trial data available in a contemporary technology format for use today. Further, a sustainable model that covers the cost of data sharing is needed, as efforts are currently being paid exclusively by industry and, in some instances, the U.S. federal government. In addition, while much of the focus on data sharing has thus far been on industry^35,36^, many other entities, particularly academia, also generate clinical research data. Lastly, there is a need going forward for systematic adoption of data format standards^37^, expectations for how long shared data will be made available, along with informed consent language, to facilitate data sharing. Publicly-available informed consent templates that explicitly allow for the sharing of data with external researchers are already available (http://mrctcenter.org/projects/informed-consent/).

The goal of data sharing initiatives should be to ensure that the data are used to conduct high-quality and rigorous research that honors the voluntary efforts of patients that participated in the trials and serves the best interests of science and public health. The research community and society are likely to greatly benefit from these secondary research efforts. With the continuous advancement of data sharing efforts, the YODA Project’s experience and the experiences of its first two data generator partners, Medtronic, Inc. and Johnson & Johnson, can be used to enhance other ongoing or future initiatives.

## Methods

We provide an overview of the history of the YODA Project, including a review of the policy and procedures iteratively developed to guide granting qualified public access to clinical trial data provided by partnering data generators. We base the review on the experience with the first two partners in the Project, Medtronic, Inc. and Johnson & Johnson. This policy and set of procedures address the research proposal requirements, data receipt, data analysis, and dissemination of results (http://yoda.yale.edu/policies-procedures-guide-external-investigator-access-clinical-trial-data). Specifically, the policy guides the procedures that are used to make clinical trial data (including both Clinical Study Reports [CSRs] and participant-level trial data) available to external investigators for independent scientific examination. Key aspects of the policy and the underlying decisions were informed by the following:

- The YODA Project’s core principles of fairness and transparency;
- The YODA Project’s review of the literature and policies from other organizations engaged in clinical trial data sharing;
- Recommendations from the YODA Project Steering Committee, an independent group of leaders in the fields of clinical research and biomedical ethics assembled by the YODA Project to provide guidance;
- Recommendations from other experts in the field, general public, and industry partners through a public comment period and personal communication; and
- The YODA Project’s accumulated experience with sharing participant-level clinical trial data.

Where appropriate, descriptive statistics were used to characterize the status of data requests and approvals.

## Additional information

**How to cite this article**: Ross, J. S. *et al.* Overview and experience of the YODA Project with clinical trial data sharing after 5 years. *Sci. Data.* 5:180268 doi: 10.1038/sdata.2018.268 (2018).

**Publisher’s note**: Springer Nature remains neutral with regard to jurisdictional claims in published maps and institutional affiliations.

## Figures and Tables

**Table 1:** Details of YODA Project inquiry process for Johnson & Johnson clinical trials as of August 27, 2018.

Total inquiries, No.	161
Total inquiries answered to date, No. (%)	159 (98.8%)
Inquiry led to full data request, No. (%)	31 (19.3%)
Median number of days for response to inquiry (Interquartile Range)	15 (7.5–41.5)
Total unique trials requested within answered inquiries, No.	207
Trial data can be made “available” to request, No. (%)	124 (59.9%)
Trial data cannot be made “available” to request, No. (%)	83 (40.1%)
Regulatory approval not yet received, No. (%)	17 (20.5%)
Trial ongoing or completed<18 months ago, No. (%)	26 (31.3%)
Data cannot be adequately de-identified, No. (%)	0 (0%)
Partner of Data Holder has not agreed to share, No. (%)	11 (13.3%)
Trial is out of scope (i.e., Phase 1, OTC, etc.), No. (%)	25 (30.1%)
Data subject to partner agreement; researcher advised to contact partnering Data Holder, No. (%)	2 (2.4%)
Data cannot be converted to electronic format, No. (%)	1 (1.2%)
Trial materials not available in English, No. (%)	5 (6.0%)

**Table 2:** Details of Johnson & Johnson clinical trials available to request as of August 27, 2018.

Trials Available, No.	270
Products Available, No.	31
Trial Enrollment Size	
Mean	412.7
Median	322
Min	5
Max	2051
Sex, No. (%)	
> 50% Female	101 (37.4%)
≤ 50% Female	131 (48.5%)
[Unknown Sex]	38 (14.1%)
Race, No. (%)	
> 50% White	145 (53.7%)
≤ 50% White	26 (9.6%)
[Unknown Race]	99 (36.7%)
Mean/Median Enrollment Age, No. (%)	
0–19	24 (8.9%)
20–39	73 (27.0%)
40–59	100 (37.0%)
60+	32 (11.9%)
[Unknown Age]	41 (15.2%)
Available Data and Documentation, No. (%)	
Collected datasets	246 (91.1%)
Analysis datasets	5 (1.9%)
[No participant-level data]	19 (7.0%)
Clinical study report (CSR)	252 (93.3%)
Protocol with amendments	256 (94.8%)
Statistical analysis plan	243 (90.0%)
Annotated case report form	224 (83.0%)
Data definition specification	194 (71.9%)
CSR summary available on site	187 (69.3%)
CSR summary not yet prepared	66 (24.4%)

**Table 3:** Therapeutic areas of Johnson & Johnson clinical trials available to request as of August 27, 2018.

Behaviors and Mental Disorders, No. (%)	106 (39.3)
Muscle, Bone, and Cartilage Diseases, No. (%)	26 (9.6)
Digestive System Diseases, No. (%)	23 (8.5)
Cancers and Other Neoplasms, No. (%)	19 (7.0)
Nutritional and Metabolic Diseases, No. (%)	18 (6.7)
Skin and Connective Tissue Diseases, No. (%)	17 (6.3)
Viral Diseases, No. (%)	14 (5.2)
Blood and Lymph Conditions, No. (%)	13 (4.8)
Nervous System Diseases, No. (%)	12 (4.4)
Immune System Diseases, No. (%)	7 (2.6)
Mouth and Tooth Diseases, No. (%)	4 (1.5)
Urinary Tract, Sexual Organs, and Pregnancy Conditions, No. (%)	4 (1.5)
Respiratory Tract (Lung and Bronchial) Diseases, No. (%)	3 (1.1)
Parasitic Diseases, No. (%)	2 (0.7)
Heart and Blood Diseases, No. (%)	1 (0.4)
Neurosciences, No. (%)	1 (0.4)

**Table 4:** Conditions studied in Johnson & Johnson clinical trials available to request as of August 27, 2018.

Acne Vulgaris, No. (%)	1 (0.4)
AIDS, No. (%)	1 (0.4)
Alopecia, Androgenetic, No. (%)	2 (0.7)
Alzheimer Disease, No. (%)	23 (8.5)
Anemia, No. (%)	13 (4.8)
Arthritis, Juvenile, No. (%)	2 (0.7)
Arthritis, Psoriatic, No. (%)	2 (0.7)
Arthritis, Rheumatoid, No. (%)	17 (6.3)
Asthma, No. (%)	1 (0.4)
Atrial Fibrillation, No. (%)	1 (0.4)
Attention Deficit and Disruptive Behavior Disorders, No. (%)	4 (1.5)
Attention Deficit Hyperactivity Disorder, No. (%)	13 (4.8)
Autistic Disorder, No. (%)	2 (0.7)
Bipolar Disorder, No. (%)	20 (7.4)
Colitis, Ulcerative, No. (%)	10 (3.7)
Conduct Disorder, No. (%)	2 (0.7)
Contraception, No. (%)	2 (0.7)
Critical Illness, No. (%)	2 (0.7)
Crohn’s Disease, No. (%)	13 (4.8)
Dementia, No. (%)	4 (1.5)
Dentinal Hypersensitivity, No. (%)	4 (1.5)
Depressive Disorder, Major, No. (%)	2 (0.7)
Dermatitis, Atopic, No. (%)	1 (0.4)
Diabetes Mellitus, Type 2, No. (%)	14 (5.2)
Epilepsy, No. (%)	1 (0.4)
Healthy Volunteers, No. (%)	1 (0.4)
Helminth Infections, No. (%)	1 (0.4)
Hepatitis C, No. (%)	11 (4.1)
HIV Infections, No. (%)	6 (2.2)
Hypertension, No. (%)	1 (0.4)
Leukemia, No. (%)	1 (0.4)
Liposarcoma, Myxoid, No. (%)	1 (0.4)
Liposarcoma/Leiomyosarcoma, Advanced, No. (%)	2 (0.7)
Migraine Disorders, No. (%)	9 (3.3)
Multiple Myeloma, No. (%)	6 (2.2)
Neoplasms, No. (%)	8 (3)
Neoplasms, Breast, No. (%)	1 (0.4)
Neoplasms, Ovarian, No. (%)	2 (0.7)
Neoplasms, Prostatic, No. (%)	2 (0.7)
Obesity, No. (%)	5 (1.9)
Partial Seizure Disorder, No. (%)	1 (0.4)
Psoriasis, No. (%)	14 (5.2)
Psychosis, No. (%)	1 (0.4)
Pyelonephritis, No. (%)	1 (0.4)
Rabies, No. (%)	3 (1.1)
Sarcoma, No. (%)	6 (2.2)
Schizoaffective Disorder, No. (%)	3 (1.1)
Schizophrenia, No. (%)	38 (14.1)
Seizures, No. (%)	1 (0.4)
Solid Tumor, No. (%)	1 (0.4)
Spondylitis, Ankylosing, No. (%)	5 (1.9)
Tuberculosis, No. (%)	2 (0.7)
Uni-polar Depression, No. (%)	1 (0.4)
Urinary Tract Infections, No. (%)	1 (0.4)
Vaginitis Infectious Vaginosis, No. (%)	1 (0.4)
Venous Thrombosis, No. (%)	1 (0.4)

**Table 5:** Product names of Johnson & Johnson clinical trials available to request as of August 27, 2018.

RISPERDAL®, No. (%)	25 (9.3)
INVEGA®, No. (%)	23 (8.5)
TOPAMAX®, No. (%)	22 (8.1)
REMICADE®, No. (%)	21 (7.8)
SIMPONI®, No. (%)	21 (7.8)
RAZADYNE®, No. (%)	20 (7.4)
STELARA®, No. (%)	19 (7.0)
INVOKANA®, No. (%)	13 (4.8)
PROCRIT®, No. (%)	13 (4.8)
CONCERTA®, No. (%)	12 (4.4)
INVEGA SUSTENNA®, No. (%)	11 (4.1)
OLYSIO®, No. (%)	11 (4.1)
RISPERDAL CONSTA®, No. (%)	10 (3.7)
YONDELIS®, No. (%)	10 (3.7)
DARZALEX®, No. (%)	5 (1.9)
Other, No. (%)	5 (1.9)
Mouth Rinse, potassium oxalate 1.4%, No. (%)	4 (1.5)
MONONESSA ® ORTHO-CYCLEN ® ORTHO TRI-CYCLEN ® TRINESSA ®, No. (%)	3 (1.1)
PREZISTA®, No. (%)	3 (1.1)
DOXIL®, No. (%)	2 (0.7)
EDURANT®, No. (%)	2 (0.7)
PLIVENSIA™, No. (%)	2 (0.7)
Rogaine 5% Women’s Foam, No. (%)	2 (0.7)
SIRTURO®, No. (%)	2 (0.7)
VERMOX®, No. (%)	2 (0.7)
ZYTIGA®, No. (%)	2 (0.7)
INTELENCE®, No. (%)	1 (0.4)
LEVAQUIN®, No. (%)	1 (0.4)
TERAZOL ®, No. (%)	1 (0.4)
THERMOCOOL® SMARTTOUCH™ Catheter, No. (%)	1 (0.4)
TREMFYA®, No. (%)	1 (0.4)

**Table 6:** Product class of Johnson & Johnson clinical trials available to request as of August 27, 2018.

Atypical Antipsychotics, No. (%)	69 (25.6)
Antirheumatic Agents - Biologic Response Modifiers, No. (%)	50 (18.5)
Anticonvulsants, No. (%)	22 (8.1)
Alzheimer’s Disease - Cholinesterase Inhibitors, No. (%)	20 (7.4)
Antiviral Agents, No. (%)	17 (6.3)
Antipsoriatics, No. (%)	14 (5.2)
Stimulants/ADHD/Anorexiants, No. (%)	12 (4.4)
Antineoplastic Agents, No. (%)	10 (3.7)
Colony-Stimulating Factors, No. (%)	9 (3.3)
Diabetes Related- Other, No. (%)	9 (3.3)
Monoclonal Antibody, No. (%)	5 (1.9)
Hematologic Agents, No. (%)	4 (1.5)
Mouth Rinse Device, No. (%)	4 (1.5)
Sodium-Glucose Co-Transporter 2 (SGLT2) Inhibitor, No. (%)	4 (1.5)
Immunizations, No. (%)	3 (1.1)
OB/GYN, No. (%)	3 (1.1)
Antimycobacterial Agents, No. (%)	2 (0.7)
Antiparasitics, No. (%)	2 (0.7)
Hormones, No. (%)	2 (0.7)
Oncology - Antibiotic, No. (%)	2 (0.7)
Other, No. (%)	2 (0.7)
Skin & Mucous Membrane Agents, Miscellaneous, No. (%)	2 (0.7)
Cardiovascular Devices, No. (%)	1 (0.4)
Dermatology, No. (%)	1 (0.4)
Quinolones - 3rd gen., No. (%)	1 (0.4)

**Table 7:** Details of data requests received for Johnson & Johnson clinical trials as of August 27, 2018.

Trials Available, No.	270
Trials Shared as Part of Approved Requests, No. (%)	183 (67.8)
Complete Data Requests Received, No.	100
Requests Requiring Revision During Review, No. (%)	36 (36.0)
Purpose of Proposed Research	
New research on treatment effectiveness or safety, No. (%)	57 (57.0)
Meta-analysis, No. (%)	45 (45.0)
Validating previous research on treatment effectiveness or safety, No. (%)	24 (24.0)
Research on clinical prediction or risk prediction, No. (%)	20 (20.0)
Develop or refine statistical methods, No. (%)	13 (13.0)
Research on clinical trial methods, No. (%)	12 (12.0)
Preliminary research for a grant proposal, No. (%)	10 (10.0)
Research on comparison group, No. (%)	6 (6.0)
Data Requests Approved, No. (%)	90 (90.0)
Data Requests Under Review, No. (%)	2 (2.0)
Data Requests Withdrawn/Closed, No. (%)	8 (8.0)
Requested data could not be used to address research question, No.	2
Data could not be downloaded as requested by investigator, No.	3
Investigator did not respond to YODA Project request for additional clarification, No.	2
Investigator withdrew approved request prior to signing DUA due to lack of resources, No.	1
Requests with Data Access [DUA signed by both parties], No. (%)	82 (82.0)
Requests with Publications, No.	11

**Table 8:** Publications using data made available through the YODA Project.

First Author	Publication Title	Journal	Year	Publication ID	Cited by:
Fu, R	Effectiveness and harms of recombinant human bone morphogenetic protein-2 in spine fusion: a systematic review and meta-analysis.	*Ann Intern Med*	2013	doi:10.7326/0003-4819-158-12-201306180-00006	299
Simmonds, MC	Safety and effectiveness of recombinant human bone morphogenetic protein-2 for spinal fusion: a meta-analysis of individual-participant data.	*Ann Intern Med*	2013	doi:10.7326/0003-4819-158-12-201306180-00005	233
Laurie, AL	Meta-analysis of the Impact of Patient Characteristics on Estimates of Effectiveness and Harms of Recombinant Human Bone Morphogenetic Protein-2 in Lumbar Spinal Fusion.	*Spine*	2016	doi:10.1097/BRS.0000000000001580	3
Noshchenko, A	What Is the Clinical Relevance of Radiographic Nonunion After Single-Level Lumbar Interbody Arthrodesis in Degenerative Disc Disease? A Meta-Analysis of the YODA Project Database.	*Spine*	2016	doi:10.1097/BRS.0000000000001113	5
Mospan, GA	5-Day versus 10-Day Course of Fluoroquinolones in Outpatient Males with a Urinary Tract Infection (UTI).	*J Am Board Fam Med*	2016	doi:10.3122/jabfm.2016.06.160065	4
Storgaard, H	Benefits and Harms of Sodium-Glucose Co-Transporter 2 Inhibitors in Patients with Type 2 Diabetes: A Systematic Review and Meta-Analysis.	*PLoS One*	2016	doi:10.1371/journal.pone.0166125	37
Gay, HC	Feasibility, Process, and Outcomes of Cardiovascular Clinical Trial Data Sharing: A Reproduction Analysis of the SMART-AF Trial.	*JAMA Cardiol*	2017	doi:10.1001/jamacardio.2017.3808	6
Corbett, M	Certolizumab pegol and secukinumab for treating active psoriatic arthritis following inadequate response to disease-modifying antirheumatic drugs: a systematic review and economic evaluation.	*Health Technol Assess*	2017	doi:10.3310/hta21560	4
Mbuagbaw, L	Review of available evidence on the use of bedaquiline for the treatment of multidrug-resistant tuberculosis: Data analysis report; Appendix to A 2016 review of available evidence on the use of bedaquiline in the treatment of multidrug-resistant tuberculosis.	World Health Organization	2017	Report No. WHO/HTM/TB/2017.01	2
Wang, R	Comparative Efficacy of Tumor Necrosis Factor-alpha Inhibitors in Ankylosing Spondylitis: A Systematic Review and Bayesian Network Metaanalysis.	*J Rheumatol*	2018	doi:10.3899/jrheum.170224	1
Schneider-Thoma J	Second-generation antipsychotic drugs and short-term mortality: a systematic review and meta-analysis of placebo-controlled randomised controlled trials.	*Lancet Psychiatry*	2018	doi: 10.1016/S2215-0366(18)30177-9	1
Singh, S	Impact of Obesity on Short- and Intermediate-Term Outcomes in Inflammatory Bowel Diseases: Pooled Analysis of Placebo Arms of Infliximab Clinical Trials.	*Inflamm Bowel Dis*	2018	doi:10.1093/ibd/izy135	
Singh, S	No Benefit of Concomitant 5-Aminosalicylates in Patients With Ulcerative Colitis Escalated to Biologic Therapy: Pooled Analysis of Individual Participant Data From Clinical Trials.	*Am J Gastroenterol*	2018	doi:10.1038/s41395-018-0144-2	
Singh, S	Obesity and Response to Infliximab in Patients with Inflammatory Bowel Diseases: Pooled Analysis of Individual Participant Data from Clinical Trials.	*Am J Gastroenterol*	2018	doi:10.1038/s41395-018-0104-x	
Zou, X	The role of PANSS symptoms and adverse events in explaining the effects of paliperidone on social functioning: a causal mediation analysis approach.	*NPJ Schizophrenia*	2018	doi:10.1038/s41537-018-0054-8	
Spertus, J	Risk of weight gain for specific antipsychotic drugs: a meta-analysis.	*NPJ Schizophrenia*	2018	doi:10.1038/s41537-018-0053-9	

## Supporting documentation for each clinical trial made publicly available on the YODA Project webpages.

- o Study Title
- o Sponsor Protocol Number
- o Product Name
- o Generic Name
- o Therapeutic Area
- o Product Class
- o Condition(s) Studied
- o ClinicalTrials.gov NCT Number and Link to Record
- o Link to PubMed Primary Publication Record
- o Study Phase
- o Enrollment
- o Mean/Median Age
- o % Female
- o % White
- o Study Synopsis Link
- o Availability (yes/no) of annotated case report forms, data definition specifications, protocol with amendments, analysis datasets, statistical analysis plans, and full clinical study reports

## Information required to be submitted as part of any routine data request.

- o Investigator name, affiliation, key personnel
- o Narrative summary
- o Public abstract
- o Research proposal, which includes a clear description of the project background, research objectives, and proposed methods, such as the study design, pre-specification of the sample inclusion/exclusion criteria, main outcome measures and statistical analysis plan
- o Conflict of interest statement
- o Timeline and dissemination plan
